# The Lin28/Let-7 System in Early Human Embryonic Tissue and Ectopic Pregnancy

**DOI:** 10.1371/journal.pone.0087698

**Published:** 2014-01-31

**Authors:** Teresa Lozoya, Francisco Domínguez, Antonio Romero-Ruiz, Liliana Steffani, Sebastián Martínez, Mercedes Monterde, Blanca Ferri, Maria Jose Núñez, AinhoaRomero-Espinós, Omar Zamora, Marta Gurrea, Susana Sangiao-Alvarellos, Olivia Vega, Carlos Simón, Antonio Pellicer, Manuel Tena-Sempere

**Affiliations:** 1 Hospital Universitario La Fe, Valencia, Spain; 2 INCLIVA Biomedical Research Institute, Valencia, Spain; 3 Universidad de Córdoba; CIBER Obesidad y Nutrición (CIBERobn) & IMIBIC/HURS, Córdoba, Spain; 4 Fundación Instituto Valenciano de Infertilidad (FIVI) and Instituto Universitario IVI/INCLIVA, Valencia University, Valencia, Spain; 5 Fundación Instituto Valenciano de Infertilidad (FIVI) and Instituto Universitario IVI/INCLIVA, Valencia University, Valencia, Spain and Department of Obstetrics and Gynecology, School of Medicine, Stanford University, California, United States of America; VU University Medical Center, Netherlands

## Abstract

Our objective was to determine the expression of the elements of the Lin28/Let-7 system, and related microRNAs (miRNAs), in early stages of human placentation and ectopic pregnancy, as a means to assess the potential role of this molecular hub in the pathogenesis of ectopic gestation. Seventeen patients suffering from tubal ectopic pregnancy (cases) and forty-three women with normal on-going gestation that desired voluntary termination of pregnancy (VTOP; controls) were recruited for the study. Embryonic tissues were subjected to RNA extraction and quantitative PCR analyses for LIN28B, Let-7a, miR-132, miR-145 and mir-323-3p were performed. Our results demonstrate that the expression of LIN28B mRNA was barely detectable in embryonic tissue from early stages of gestation and sharply increased thereafter to plateau between gestational weeks 7–9. In contrast, expression levels of Let-7, mir-132 and mir-145 were high in embryonic tissue from early gestations (≤6-weeks) and abruptly declined thereafter, especially for Let-7. Opposite trends were detected for mir-323-3p. Embryonic expression of LIN28B mRNA was higher in early stages (≤6-weeks) of ectopic pregnancy than in normal gestation. In contrast, Let-7a expression was significantly lower in early ectopic pregnancies, while miR-132 and miR-145 levels were not altered. Expression of mir-323-3p was also suppressed in ectopic embryonic tissue. We are the first to document reciprocal changes in the expression profiles of the gene encoding the RNA-binding protein, LIN28B, and the related miRNAs, Let-7a, mir-132 and mir-145, in early stages of human placentation. This finding suggests the potential involvement of LIN28B/Let-7 (de)regulated pathways in the pathophysiology of ectopic pregnancy in humans.

## Introduction

Ectopic pregnancy is an important cause of maternal morbidity and mortality whose etiology is still unknown. It is a condition in which a fertilized ovum does not implant into the uterine cavity, being the most common site of ectopic implantation the Fallopian tube [1]. Its incidence is 1–2% of all reported pregnancies [2], and it is the main cause of maternal death in early stages of pregnancy (4–10% of deaths related to pregnancy) [3] . Given the impact of this pregnancy disease in reproductive success and maternal outcomes, and considering of the conspicuous lack of accurate and early markers [3], a better understanding of the molecular mechanisms involved in this condition is eagerly needed. To accomplish this goal, deepening of our physiological knowledge of the early events of normal human placentation becomes also mandatory.

Recent evidence has conclusively demonstrated that the regulation of numerous key biological processes does not depend only of classical transcriptional mechanisms, and other regulatory phenomena, such as epigenetic mechanisms, do have important roles [4]. These epigenetic mechanisms include not only DNA methylation and the post-translational modifications of histones, but also small non-coding RNAs, including microRNAs [4], [5].

MicroRNAs (miRNAs) are small RNA fragments (18 to 25 nucleotides) that do not encode proteins but act as post-transcriptional regulators of many gene targets [6], [7]. The number of known miRNAs in different species, including humans, has exponentially increased in recent years [8], and to date, nearly one thousand individual miRNAs have been identified in humans. The interest in this class of regulatory molecules has also dramatically expanded recently, as they have been shown to be involved in the regulation of key biological phenomena, including cellular stemness, proliferation, apoptosis and differentiation [9], while their deregulation is thought to play important pathogenic roles in multiple diseases, including prominently cancer, as well as sepsis, autoimmune diseases and some cardiovascular and metabolic disorders [10].

The main mechanism used by miRNAs for gene regulation is related with their binding capacity to complementary sequences of RNAs, mainly located in the 3′-untranslated regions, causing (in most cases) gene silencing. A remarkable feature of miRNAs is that they are rather stable and detectable in circulation and other body fluids, thus opening up the possibility of their use as biomarkers of specific disease conditions [10]. Indeed, the recent identification of circulating pregnancy-associated microRNAs has raised considerable interest, as they may help in the early diagnosis (and the eventual etiopathogenic characterization) of complications related to pregnancy [9], [11]–[13].

The family of *Let-7* is a numerous group of related miRNAs, encoded by various gene clusters, which is among the most highly expressed in mammals [14]; in humans, up to 11 different *let-7* members encoded by 4 gene clusters have been identified. MiRNAs of the *Let-7* family have been shown to participate in the control of cellular pluripotency, proliferation and differentiation [15], [16]. Besides their key roles during development and gametogenesis, *Let-7* miRNAs have been cataloged as tumor suppressors, whose deregulation may lead to tumorigenesis [15], [16]. In fact, different oncogenes, such as such as c-Myc, Ras and Cdk6 are known targets of *Let-7* miRNAs.

LIN28 is a RNA binding-protein that is thought to play important roles during development [17], pubertal maturation [18]–[20], and in the control of spermatogonial stemness [21], [22], among other functions. Two LIN28-related genes, namely *LIN28* (or *LIN28A*) and *LIN28B*, are found in mammals [23]–[25]. LIN28 proteins are extensively distributed during mammalian embryonic maturation, but its expression becomes restricted to some tissues in adulthood [26]. LIN28 proteins are capable of binding the precursors of *Let-7* miRNAs, thus blocking their capacity to interact with the miRNA-maturing enzyme, Dicer, and preventing their processing into mature miRNAs [17]. In turn, *Let-7* miRNAs participate in the regulation of LIN28 expression, which is controlled also by other upstream elements, such as myc, and, according to bioinformatics predictions, other miRNAs, as *mir-132* and *mir-145*, thus forming a complex regulatory hub that is involved in different processes [19].

In spite of their paramount importance and the concurrence of complex developmental phenomena, the roles of miRNA regulatory pathways in human placentation and early pregnancy remain largely unknown [27], even though placental formation resembles features of tumor progression and invasiveness; phenomena in which miRNAs have been shown to play important regulatory roles. Nonetheless, significant progress in our knowledge of the involvement of miRNAs in human pregnancy has occurred recently, as various pregnancy-associated miRNAs clusters have been identified [9]. Moreover, a number of circulating, pregnancy-specific miRNAs have been reported; a finding that has drawn considerable interest as these may serve as potential biomarkers of different gestational complications [11], [27]. Yet, a majority of the expression studies published have focused in late stages of pregnancy, and little attention has been paid so far to the characterization of the expression patterns of specific miRNAs in early phases of human placental formation. As illustrative example, the expression of *Let-7* miRNAs and LIN28 has been recently reported in human gestational tissues at term [28]. However, no information regarding the expression of the elements of the LIN28/Let-7 system in human placenta at early stages of gestation is available. Similarly, despite very recent findings on circulating *mir-323-3p* as putative biomarker of ectopic pregnancy [29], to our knowledge, no information has been so far reported on changes of expression in ectopic placental tissue of miRNAs in general, and of the elements of the LIN28/Let-7 system in particular.

In this context, the aim of this work is to evaluate the expression patterns of *LIN28B* mRNA, as well as the related *Let-7*, *mir-132* and *mir-145*, in human embryonic tissue from early gestation, between weeks 5 and 9 of amenorrhea. In addition, changes in the expression of the above targets were explored in embryonic material from ectopic pregnancies, at this early gestational window. For comparative purposes, the embryonic expression levels of the recently proposed circulating biomarker of ectopic pregnancy, *mir-323-3p* [29], were also assayed in normal and ectopic gestations.

## Materials and Methods

### Ethical Approval

The present study was approved by the Institutional Review Board/Independent Ethics Committee of the Hospital Universitario La Fe, Valencia, Spain. Early embryonic tissue (mostly trophoblast) was collected after obtaining the corresponding informed consent from each patient.

### Human Samples

Forty-three women with a normal ongoing pregnancy that desired a voluntary termination of pregnancy (VTOP) and seventeen patients suffering from tubal ectopic pregnancy were recruited (**Table 1**). Patients were diagnosed of suspected ectopic pregnancy by using transvaginal ultrasound combined with serial determinations of blood BhCG. The patients provided the date of their last menstrual period. Diagnostic confirmation and treatment were done by laparoscopy; laparoscopic procedure was elected as the patients did not meet criteria for medical treatment, namely: hemodynamic instability, hemo-peritoneum, diameter of ectopic pregnancy larger than 4 cm, presence of heartbeat, severe pain, BhCG concentrations greater than 5000 UI/L [2]. None of them had received pre-operatively any medical treatment with methotrexate.

**Table 1:** Characteristics of the ectopic pregnancy (EP) patients and VTOP controls participating in this study.

	Age	Gravidity	Numberof births	History of previousspontaneous of inducedabortions	Number ofabortions	History of previousectopic pregnancy	History of abdominalor pelvic surgery	History ofinfertility	IUD user or progestagencontraception	Smoking
**EP**	30,9	2,1	0,5	35,3%	0,5	11,8%	35,3%	11,6%	5,9%	23,5%
**Controls**	21,1	2	0,5	38,2%	0,4	2,9%	11,8%	0%	2,9%	47,1%

After signing the informed consent to participate in the study and for the intervention, a laparoscopic procedure was performed, using conventional techniques. Ectopic pregnancies selected for this study were unruptured gestations located in the isthmus or the proximal ampulla. The tube containing the ectopic pregnancy was grasped at both sites (approximately 1 cm away from the gestation) and bipolar coagulation applied. Similarly, the adjacent mesentery was also coagulated. Then, salpingectomy was performed employing scissors. One of the investigators present in the operating room (TL) perform a longitudinal incision to the anti mesentery surface of the tube and applied a mild pressure with two fingers to extract the gestational sac. Embryonic tissue was carefully separated from obvious blood clots and tubal tissue in the operating room under a stereomicroscope. Embryonic samples were immediately placed in TRIzol reagent (*see below*), frozen and stored at −80°C until use. A piece of sample was sent to the Pathology Department (Hospital Universitario La Fe), which provided histological confirmation of ectopic pregnancy and absence of tubal tissue. Fetal dilation and evacuation method or fetal aspiration technique were performed in VTOP women to obtain Embryonic tissue. Tissue samples were immediately stored at −80°C until use.

### RNA Extraction and Quantitative PCR

Total RNA was isolated from human embryonic samples from different stages of pregnancy (both control and ectopic gestations), using TRIzol reagent (Invitrogen, CA), following the manufacturer’s protocol. The quality and concentration of the isolated RNA were determined by spectrophotometry, following standard procedures. Real-time qPCR was performed on the samples using a Bio-Rad SFX 96 Real-Time System (Bio-Rad Laboratories, Hercules, CA), as described in detail elsewhere [19], [22].

For quantification of *LIN28B* mRNA in embryonic samples, 1 µg of total RNA per tissue sample was treated with RQ1 RNAse-free DNAse-I (Promega, Madison, WY) and retro-transcribed (RT) in a 30 µl reaction using iScript™ Reverse Transcription Super-mix (Bio-Rad Laboratories). For real-time PCR amplification, we used SYBR Green qPCR Master Mix (Promega), with the following primer sequences: hLIN28B-forward: 5′-CAT CTC CAT GAT AAA CCG AGA GG-3′; hLIN28B-reverse: 5′-GTT ACC CGT ATT GAC TCA AGG C-3′. The primer pair: hL19-forward: 5′-GAA ATC GCC AAT GCC AAC TC-3′ and hL19-reverse: 5′-ACC TTC AGG TAC AGG CTG TG-3′ was used for amplification of a 290-bp fragment of the mRNA of ribosomal L19 protein, used as internal control for reaction efficiency and sample loading.

PCR was initiated by one cycle of 95°C for 2-minutes, followed by 35 cycles of 15-seconds at 95°C, 30-seconds at 62°C, and 10-seconds at 72°C, followed by one final cycle of 72°C for 1-minute. Relative *LIN28* mRNA levels were normalized against the expression levels of L19 internal control transcript.

For miRNA analyses, expression levels of *Let-7a*, *mir-145*, *mir-132* and *mir-323-3p* were assayed, following a previously published procedure [19]. cDNA was synthesized by using 10 ng total RNA with TaqMan–specific RT primers and the TaqMan microRNA reverse transcription kit (Applied Biosystems, Foster City, CA). Thereafter, quantitative RT PCR was performed using pre-designed assays for *Let-7a*, *mir-145*, *mir-132*, *mir-323-3p* and *RNU6* (Applied Biosystems). PCR reactions were carried out as follows: 50°C for 2-minutes, 95°C for 10-minutes, followed by 40 cycles of 95°C for 15-seconds and 60°C for 1 minute. For quantitative miRNA determination, RNU6 gene served as internal control reference.

### Presentation of Data and Statistics

**Figure 1:**
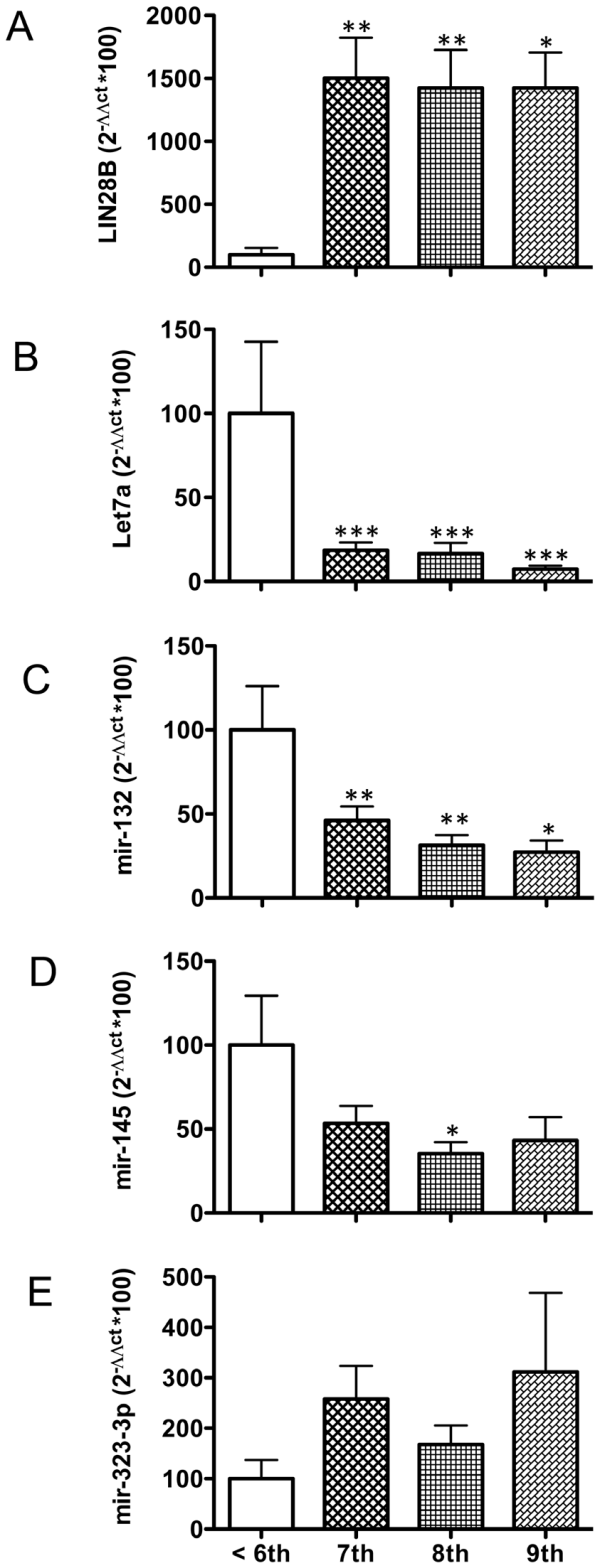
Expression profiles of LIN28B and several miRNAs in embryonic tissue during normal placentation at early gestational periods. Relative mRNA and miRNA levels were assayed in placental samples from normal pregnancies, obtained from women undergoing voluntary termination of pregnancy (VTOP) at gestational ages ranging from week-5 to week-9 after amenorrhea. Samples from week-5 and −6 of amenorrhea were grouped as ≤6-week gestational samples. Targets analyzed were: *LIN28B* (**A**), *Let-7a* (**B**), *mir-132* (**C**), *mir-145* (**D**) and *mir-323-3p* (**E**). Data are presented as mean ± SEM. Expression levels were quantitatively analyzed using the 2ΔΔCt method and were normalized to values from ≤6-week gestational samples. *, P<0.05; **, P<0.01; and ***, P<0.001 vs. values in ≤6-week gestational samples (One-way ANOVA followed by Newman-Keuls test).

**Figure 2:**
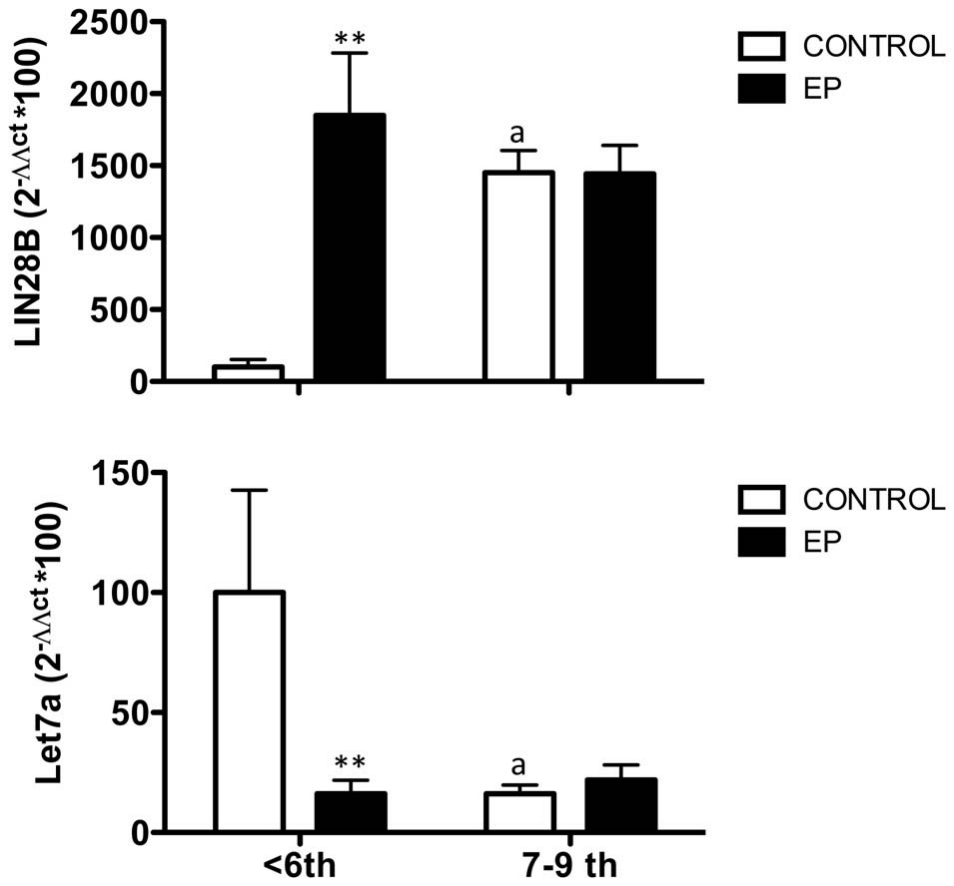
Altered expression of LIN28B and Let-7a in embryonic tissue from early ectopic pregnancies. Relative levels of *LIN28B* mRNA (**A**) and *Let-7* miRNA (**B**) were assayed in samples from VTOP controls and ectopic pregnancy patients. For presentation, the data were grouped in two gestation age ranges: ≤6-week vs. 7–9 week gestational samples. Data are presented as mean ± SEM. Expression levels were quantitatively analyzed using the 2ΔΔCt method and were normalized to values from ≤6-week VTOP (control) samples. Data were analyzed by two-way ANOVA, followed by individual Newman-Keuls tests; **, P<0.01 vs. corresponding values in control samples (effect of EP); a, P<0.01 vs. corresponding values in <6-week samples (effect of gestational age).

Hormonal data are presented as mean ± SEM. Results were statistically analyzed using one-way ANOVA (for studies on expression profiles in normal pregnancies in which the variable was the gestational age; *see* *Figure 1*) or two-way ANOVA (for comparison of expression levels as function of gestational age and ectopic placentation; *Figures 2* *–* *3*), in order to detect inter-group differences. If significant interactions were found, the data were further analyzed using post hoc comparisons (using Newman-Keuls tests), in order to identify simple effects. Statistical analyses were conducted following the procedures of the software package Prism-GraphPad (La Jolla, CA, USA). *P*-values ≤0.05 were considered statistically significant.

**Figure 3:**
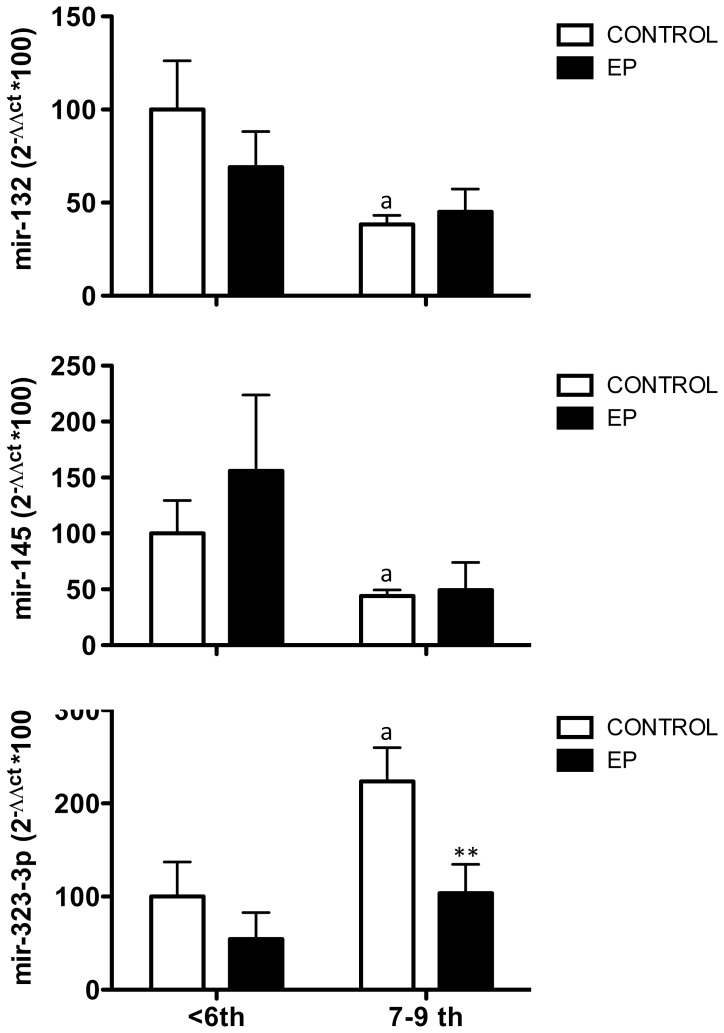
Expression profiles of mir-132, mir-145 and mir-323-3p in embryonic tissue from early ectopic pregnancies. Relative levels of *mir-132* mRNA (**A**), *mir-145* and *mir-323-3p* (**B**) were assayed in embryonic samples from VTOP controls and ectopic pregnancy patients. For presentation, the data were grouped in two gestation age ranges: ≤6-week vs. 7–9 week gestational samples. Data are presented as mean ± SEM. Expression levels were quantitatively analyzed using the 2ΔΔCt method and were normalized to values from ≤6-week VTOP (control) samples. Data were analyzed by two-way ANOVA, followed by individual Newman-Keuls tests; **, P<0.01 vs. corresponding values in control samples (effect of EP); a, P<0.01 vs. corresponding values in <6-week samples (effect of gestational age).

## Results

### Embryonic Tissue Expression of LIN28B and Related miRNAs during Early Human Gestation

Embryonic tissues from early stages of normal gestation (between 5- and 9-weeks after amenorrhea) were studied. Given the limited number of samples available, and in order to increase statistical power, samples from 5- and 6-weeks were grouped for analysis as ≤6-weeks samples. Investigation of the relative mRNA levels of the RNA-binding protein, *LIN28B*, in early human embryonic tissue revealed nearly negligible expression in ≤6-week placentas, with a sharp increase of *LIN28B* mRNA levels at week-7 and plateau levels thereafter up to week-9 (**Figure 1A**). In clear contrast, *Let-7a* miRNA expression was maximal in ≤6-week embryonic tissue, and abruptly dropped in the transition to week-7, to remain low in expression up to week-9 (**Figure 1B**). A similar profile was observed for *mir-132* and *mir-145*, although the decline in expression was more gradual and less pronounced (e.g., on week-7, mean *Let-7* levels were reduced in >80%, while *mir-132* and *mir-145* levels were nearly halved), and in the case of *mir-145* only reached statistical significance on week-8 (**Figure 1C–D**). On the other hand, an inverse trend was detected for *mir-323-3p*, whose expression levels were minimal in ≤6-week embryonic tissue, and increased thereafter, with maximal mean levels at week-9. Yet, due to some variability within groups, these differences did not reach statistical significance when compared on a weekly basis (**Figure 1E**).

### Embryonic Tissue Expression of LIN28B and Related miRNAs in Ectopic Pregnancies

The expression levels of the above targets were comparatively analyzed in embryonic tissues from ectopic pregnancies, during this early developmental window. While samples were assayed individually, in order to increase the statistical power of our analyses, values were grouped into ≤6-week vs. 7–9 week samples for comparison. This procedure allowed to increase the number of individual determinations per group and was based on the trends observed from individual sample analyses.

Grouped analysis of the expression values confirmed that *LIN28B* mRNA expression increases (by nearly 4-fold) in normal embryonic tissue between ≤6-week and later stages of early gestation. Notably, *LIN28B* mRNA levels in embryonic tissue from ≤6-week ectopic pregnancy were significantly higher than in the control group, while this difference obliterated in 7–9 week samples (**Figure 2A**). Quite oppositely, we detected a substantial drop in *Let-7* miRNA levels in normal embryonic tissue between ≤6-week and later stages of gestation (in keeping with individual analyses), while in ectopic pregnancies, *Let-7a* miRNA expression was already very low in ≤6-week samples (**Figure 2B**).

Similar analyses were conducted for the other miRNA targets. Expression levels of *mir-132* and *mir-145* declined between ≤6-week and later stages of early gestation, and these profiles were not substantially altered in ectopic gestation (**Figure 3A–B**), although a non-significant trend for higher levels of *mir-145* in ≤6-week ectopic embryonic tissue was detected. In contrast, *mir-323-3p* expression levels raised in normal human embryonic tissue between ≤6-week and later stages of early gestation; a difference that was statistically significant due to the grouped analysis of the samples (≤6-week vs. 7–9 week groups). Interestingly, *mir-323-3p* levels were similar in normal and ectopic gestation at ≤6-weeks; yet, the rise of expression detected at later stages of normal early gestation was not observed in ectopic pregnancies, so that *mir-323-3p* levels in 7–9 week ectopic embryonic tissue were significantly lower than in the control group (**Figure 3C**).

## Discussion

Ectopic pregnancy is a rather common condition that represents the major cause of maternal death during early stages of pregnancy, for which improved diagnostic tools are eagerly needed. In current practice, diagnosis of ectopic pregnancy requires multiple clinic visits [30], and quite often needs laparoscopy as method of certainty, with the risks associated to this intervention and the negative maternal psychological and reproductive consequences that it entails. These above reasons justify the active search for non-invasive, accurate methods for diagnosis of ectopic pregnancy, whose identification should be based in a better understanding of the early stages of human placentation and the pathophysiological mechanisms underlying this disease.

Considering that placental formation displays features that resembles those of tumor progression and invasiveness, as well as complex developmental processes (phenomena in which miRNA regulatory pathways seem to play an essential role), it is reasonable to hypothesize that specific miRNAs might be involved in the regulation of placentation and pregnancy in humans and other species. Indeed, a number of studies have addressed the roles of miRNAs in human gestation [9], [11], [27], and specific pregnancy-associated miRNA-clusters and circulating miRNAs have been identified, which pose obvious pathophysiological interest and may pave the way for the identification of improved biomarkers of gestational complications. On the latter, however, little attention have been paid so far to ectopic pregnancy, and to our knowledge only one study have been reported on the potential usefulness of circulating *mir-323-3p* as biomarker of ectopic pregnancy [29]. Yet, this observation has not been independently confirmed. Likewise, no comparative reports on the patterns of early embryonic tissue expression of specific miRNAs during early normal gestation and ectopic pregnancy have been presented to date. These studies may help to elucidate key pathogenic mechanisms and would illuminate the search for additional miRNA-related biomarkers of this condition.

In this work, we evaluated the expression patterns of *LIN28B*, *Let-7a* and related miRNAs in embryonic tissues from normal and ectopic pregnancies during early stages of gestation; i.e., 5–9 weeks after amenorrhea. Embryonic tissue from ectopic gestations and matched ongoing pregnancies subjected to VTOP, as representative of normal implantation, were studied. The latter were used as controls since these pregnancies were viable, with a theoretically healthy trophoblast with normal implantation, thereby eliminating external factors such as chromosomal abnormalities, toxics, thrombophilias, and any other perturbations that might lead to a spontaneous abortion and could produce also changes in the pathways under investigation.

Our analyses unveiled that very early (≤6-week) normal embryonic tissue expresses virtually negligible levels of *LIN28B* but maximal levels of *Let-7a*, as well as *mir-132* and *mir-145*. Of note, expression of LIN28 and members of the *Let-7* family had been previously reported in the placenta of rats and humans [19], [28], but to our knowledge those analyses were restricted to gestational tissues at term and no information on early expression profiles was available. Transition between gestational week-6 to week-7 brought about a marked reciprocal change in the expression of those targets, with a robust increase in *LIN28B* mRNA expression and a significant decrease of *Let-7a*, and to a lesser extent *mir-132* and *mir-145* levels. Changes in LIN28 and *Let-7* expression have been related with tumorigenesis and cancer invasiveness. Thus, *LIN28B* over-expression has been detected in several tumors and hematological malignancies [31], suggesting a role of this molecule in (promoting) invasiveness. In turn, *Let-7* miRNAs have been reported to play role as tumor suppressor, mainly by repression of oncogenes and key regulators of various mitogenic pathways, such as Myc, Hmga2, Ras, JAK and STAT3. All in all, it might be speculated that an increase in the *LIN28B/Let-7* ratio, as observed during the 6-to-7 gestational week transition, might be associated with a pro-proliferative and invasive phenotype. It is tempting to hypothesize that this change might play a role in trophoblast invasion and placental formation during this early gestational period, through as yet unknown effector mechanism. The regulatory roles in early placentation of *mir-132* and *mir-145*, whose levels also decline during this time-window and are predicted to participate in the regulation of LIN28B synthesis [19], merit further investigation.

Tubal ectopic implantation resulted in the reversal of the expression profiles of *LIN28B* and *Let-7a* in early (≤6-week) embryonic tissue. Thus, in ectopic embryonic tissue for ≤6-week gestation *LIN28B* mRNA levels were abnormally high, and remained high in 7–9 week embryonic tissue. Conversely, *Let-7a* miRNA expression was already suppressed in ≤6-week samples from ectopic pregnancies, so that this condition anticipated the drop of expression of this miRNA that normally occurred from week-7 onwards. These expression profiles would imply an increased *LIN28B/Let-7* ratio in embryonic tissue from ≤6-week ectopic gestations. Assuming that this ratio might favor a pro-proliferative and invasive phenotype, it is tenable that such a deregulation in the LIN28/Let-7 system might contribute to the perturbation of trophoblast invasion that characterizes ectopic pregnancies. Admittedly, our analyses using homogenates of early embryonic tissue do not allow discrimination of the specific cellular sources for the expression of *LIN28B* and *Let-7a* targets. Yet, the reported reciprocal changes between *LIN28B* and *Let-7a* both during early pregnancy and in ectopic placentas strongly suggest that these two factors are co-localized, further stressing the functional relevance of this reciprocal regulation. Indeed, the major known function of LIN28B is to repress the maturation of Let-7 miRNAs [17].

As mentioned above, *mir-323-3p* is the only putative biomarker of ectopic pregnancy reported to date [29]. However, identification of this marker was done in plasma and no information regarding its expression patterns in normal and ectopic placentas is available. Our comparative analyses revealed that, in contrast to *Let-7a*, *mir-132* and *mir-145*, the embryonic expression of *mir-323-3p* increased during early pregnancy, with minimal levels being detected in ≤6-week embryonic tissue that raised thereafter. Quite unexpectedly, despite the reported increase in the circulating levels of this miRNA in ectopic pregnancy, our data evidenced that ectopic embryonic tissue actually expressed lower levels of *mir-323-3p* from week-7 onwards, so that the raise in *mir-323-3p* expression observed in normal gestation is not detected in ectopic pregnancies. Thus, although *mir-323-3p* has been proposed as pregnancy-specific miRNA (32), our results cast doubt on the placental origin of the excess of this factor in ectopic gestation. In fact, the possibility that the source of circulating *mir-323-3p* in ectopic pregnancy might not be solely the embryonic tissue has been already suggested [29], [32]; our findings support the non-embryonic origin of this miRNA and call for independent confirmation of the usefulness of plasma levels of *mir-323-3p* as universal marker of ectopic gestation.

In sum, we document herein the expression profiles of elements of the Lin28/Let-7 system, as well as related miRNAs, in embryonic tissue during early stages of normal gestation and ectopic pregnancy. Our data are the first to disclose the opposite patterns of embryonic expression of *LIN28B* and *Let-7a* during early pregnancy, with an increased ratio of *LIN28B*/*Let-7a* levels in the embryonic tissue during the 6- to 7-week transition, while ectopic embryonic tissue displayed high *LIN28B*/*Let-7a* ratios already on ≤6-week gestational ages. Given the proposed roles of LIN28 and *Let-7* members in the control of cellular proliferation and tumor invasion, its is tempting to propose that the above changes in *LIN28B* and *Let-7a* expression might play a role in the control of normal and ectopic placentation.

## References

[1] Barnhart KT (2009) Clinical practice. Ectopic pregnancy. N Engl J Med 361, 379–87. 10.1056/NEJMcp0810384 19625718

[2] Farquhar CM (2005) Ectopic pregnancy. Lancet 366, 583–91. 10.1016/S0140-6736(05)67103-6 16099295

[3] Horne AW, Duncan WC, Critchley HO (2010) The need for serum biomarker development for diagnosing and excluding tubal ectopic pregnancy. Acta Obstet Gynecol Scand 89, 299–301. 10.3109/00016340903568191 PMC2971461 20199347

[4] Wilkins-Haug L (2009) Epigenetics and assisted reproduction. Curr Opin Obstet Gynecol 21, 201–6. 10.1097/GCO.0b013e32832d7b95 19458521

[5] Maccani MA, Marsit CJ (2009) Epigenetics in the placenta. Am J Reprod Immunol 62, 78–89. 10.1111/j.1600-0897.2009.00716.x PMC2813777 19614624

[6] Ambros V (2001) microRNAs: tiny regulators with great potential. Cell 107, 823–6. 10.1016/s0092-8674(01)00616-x 11779458

[7] Prieto DM, Markert UR (2011) MicroRNAs in pregnancy. J Reprod Immunol 88, 106–11. 10.1016/j.jri.2011.01.004 21353310

[8] Zhang B, Pan X, Cobb GP, Anderson TA (2007) microRNAs as oncogenes and tumor suppressors. Dev Biol 302, 1–12. 10.1016/j.ydbio.2006.08.028 16989803

[9] Morales-Prieto DM, Ospina-Prieto S, Chaiwangyen W, Schoenleben M, Markert UR (2013) Pregnancy-associated miRNA-clusters. J Reprod Immunol 97, 51–61. 10.1016/j.jri.2012.11.001 23432872

[10] Scholer N, Langer C, Dohner H, Buske C, Kuchenbauer F (2010) Serum microRNAs as a novel class of biomarkers: a comprehensive review of the literature. Exp Hematol 38, 1126–30. 10.1016/j.exphem.2010.10.004 20977925

[11] Zhao Z, Moley KH, Gronowski AM (2013) Diagnostic potential for miRNAs as biomarkers for pregnancy-specific diseases. Clin Biochem 46, 953–60. 10.1016/j.clinbiochem.2013.01.026 23396163

[12] Kotlabova K, Doucha J, Hromadnikova I (2011) Placental-specific microRNA in maternal circulation–identification of appropriate pregnancy-associated microRNAs with diagnostic potential. J Reprod Immunol 89, 185–91. 10.1016/j.jri.2011.02.006 21513988

[13] Chim SS, Shing TK, Hung EC, Leung TY, Lau TK, et al. (2008) Detection and characterization of placental microRNAs in maternal plasma. Clin Chem 54, 482–90. 10.1373/clinchem.2007.097972 18218722

[14] Roush S, Slack FJ (2008) The let-7 family of microRNAs. Trends Cell Biol 18, 505–16. 10.1016/j.tcb.2008.07.007 18774294

[15] Boyerinas B, Park SM, Hau A, Murmann AE, Peter ME (2010) The role of let-7 in cell differentiation and cancer. Endocr Relat Cancer 17, F19–36. 10.1677/ERC-09-0184 19779035

[16] Bussing I, Slack FJ, Grosshans H (2008) let-7 microRNAs in development, stem cells and cancer. Trends Mol Med 14, 400–9. 10.1016/j.molmed.2008.07.001 18674967

[17] Viswanathan SR, Daley GQ (2010) Lin28: A microRNA regulator with a macro role. Cell 140, 445–9. 10.1016/j.cell.2010.02.007 20178735

[18] Zhu H, Shah S, Shyh-Chang N, Shinoda G, Einhorn WS, et al. (2010) Lin28a transgenic mice manifest size and puberty phenotypes identified in human genetic association studies. Nat Genet 42, 26–30. 10.1038/ng.593 PMC3069638 20512147

[19] Sangiao-Alvarellos S, Manfredi-Lozano M, Ruiz-Pino F, Navarro VM, Sanchez-Garrido MA, et al. (2013) Changes in hypothalamic expression of the Lin28/let-7 system and related microRNAs during postnatal maturation and after experimental manipulations of puberty. Endocrinology 154, 942–55. 10.1210/en.2012-2006 PMC3548186 23291449

[20] Elks CE, Perry JR, Sulem P, Chasman DI, Franceschini N, et al. (2010) Thirty new loci for age at menarche identified by a meta-analysis of genome-wide association studies. Nat Genet 42,1077–85. 10.1038/ng.714 PMC3140055 21102462

[21] Aeckerle N, Eildermann K, Drummer C, Ehmcke J, Schweyer S, et al. (2012) The pluripotency factor LIN28 in monkey and human testis: a marker for spermatogonial stem cells? Mol Hum Reprod 18, 477–88. 10.1093/molehr/gas025 PMC3457707 22689537

[22] Gaytan F, Sangiao-Alvarellos S, Manfredi-Lozano M, Garcia-Galiano D, Ruiz-Pino F, et al. (2013) Distinct expression patterns predict differential roles of the miRNA-binding proteins, Lin28 and Lin28b, in the mouse testis: studies during postnatal development and in a model of hypogonadotropic hypogonadism. Endocrinology 154, 1321–36. 10.1210/en.2012-1745 23337528

[23] Moss EG, Tang L (2003) Conservation of the heterochronic regulator Lin-28, its developmental expression and microRNA complementary sites. Dev Biol 258, 432–42. 10.1016/s0012-1606(03)00126-x 12798299

[24] Balzer E, Moss EG (2007) Localization of the developmental timing regulator Lin28 to mRNP complexes, P-bodies and stress granules. RNA Biol 4, 16–25. 10.4161/rna.4.1.4364 17617744

[25] Guo Y, Chen Y, Ito H, Watanabe A, Ge X, et al. (2006) Identification and characterization of lin-28 homolog B (LIN28B) in human hepatocellular carcinoma. Gene 384, 51–61. 10.1016/j.gene.2006.07.011 16971064

[26] Yang DH, Moss EG (2003) Temporally regulated expression of Lin-28 in diverse tissues of the developing mouse. Gene Expr Patterns 3, 719–26. 10.1016/s1567-133x(03)00140-6 14643679

[27] Morales Prieto DM, Markert UR (2011) MicroRNAs in pregnancy. J Reprod Immunol 88, 106–11. 10.1016/j.jri.2011.01.004 21353310

[28] Chan HW, Lappas M, Yee SW, Vaswani K, Mitchell MD, et al. (2013) The expression of the let-7 miRNAs and Lin28 signalling pathway in human term gestational tissues. Placenta 34,443–8. 10.1016/j.placenta.2013.02.008 23545322

[29] Zhao Z, Zhao Q, Warrick J, Lockwood CM, Woodworth A, et al. (2012) Circulating microRNA miR-323–3p as a biomarker of ectopic pregnancy. Clin Chem 58, 896–905. 10.1373/clinchem.2011.179283 PMC3694411 22395025

[30] Dart R, Ramanujam P, Dart L (2002) Progesterone as a predictor of ectopic pregnancy when the ultrasound is indeterminate. Am J Emerg Med 20, 575–9. 10.1053/ajem.2002.35460 12442232

[31] Zhou J, Ng SB, Chng WJ (2013) LIN28/LIN28B: an emerging oncogenic driver in cancer stem cells. Int J Biochem Cell Biol 45, 973–8. 10.1016/j.biocel.2013.02.006 23420006

[32] Miura K, Miura S, Yamasaki K, Higashijima A, Kinoshita A, et al.. (2010) Identification of pregnancy-associated microRNAs in maternal plasma. Clin Chem 56, 1767–71. 10.1373/clinchem.2010.147660 20729298

